# The Need of and How to Secure Medical Legislation in Texas

**Published:** 1898-06

**Authors:** H. W. Cummings

**Affiliations:** Hearne, Texas


### The Need of and How to Secure Medical Legislation in Texas.

BY H. W. CUMMINGS, M. D., HEARNE, TEXAS.

[Read before the Brazos Valley Medical Association at Calvert, Texas, May 10, 1898.]

*Gentlemen and Fellow-Members of the Brazos Valley Medical Association:*

It was reluctantly that I consented to the task of writing on this subject. It is true that it is by no means an untrammeled field, for during the past several years many abler men than I have given to our profession their theories of medical legislation. Just recently the subject was again presented for consideration by our State Association, and its deliberations, in my opinion, should receive the endorsement of the entire profession of Texas.

If a full discussion of the subject were all that was needed to guarantee the enactment of a suitable and effective law in our State, I could have contented myself with what has been said and done. But in my opinion the most important part of our work is yet undone, of which I will later speak.

First.—Does Texas need medical legislation? This question has been answered a thousand times in the affirmative, and I shall take pleasure in recording myself among the yeas.

But why do we need such legislation? The constant and rapid growth of every industry, every trade and every profession in this country has been such as to create a necessity for legislation controlling their operations, and from time to time our law makers have attempted to meet the necessity and satisfy the public's demands. However, our forefathers, when they established the Constitution and laws of this great country of ours, did not intend that law-making should interfere with the pursuits of any individual or circumscribe their personal liberties, so long as such pursuits were not injurious to the general welfare, nor interfered with the personal rights of another. There has been in this country a growing sentiment against trust and combined capital, and which is proper when these concerns attempt to monopolize the business of the country, and by crushing smaller industries inflict a common injury. But legislation against trusts should not intend to injure companies which are organized for the purpose of concentrating capital into such sums as will enable them to run such industries as we now need for the development of our country; and which could not be attempted on smaller sums such as one individual might have acquired. The same principle may be applied to our medical laws. I regret to say that some of our legislators have interpreted the efforts of our profession in this direction as being prompted with an eye to eliminate unqualified physicians from our ranks; not because they were unfit to render services to the public, but of personal jealousy arising from their competition. But I declare we are far from such motives. No association of physicians should ask for the passage of a law for their own protection and benefit unless it at the same time protects in common the welfare of the people at large. As I understand the motive of our profession, we are asking for such a law as will protect the people from the pretensions of the uneducated and dishonest man who attempts to hide from the judgment of the people by going into a profession whose technicalities and mysteries protect him from their desire to know what he is. It is true we are deeply interested in such legislation; for to raise the qualification and standard of medicine is to elevate our profession upon such a plane that soon it will be rid of these pretenders, and then its votaries will command that degree of respect and esteem which is due so noble and true a profession. But may we be far from any selfish motive in our action along this line.

We now have a law on our statutes which is almost if not completely inoperative, so far as its use to accomplish its desired aim is concerned. Each judicial district has a board of examiners appointed by the district judge, with full power invested in any two members to issue certificates to practice medicine in Texas. These boards are often appointed for political influence, rather than for medical proficiency. Many of them are indifferent or careless, and even ignorant of the very branches upon which they are to pass as examiners. They will

(many of them) grant certificates indiscriminately for the asking (and a fee of $5), turning loose upon the people men who are absolutely ignorant of medicine, while they hold a certified writing that they are qualified to practice medicine and surgery, in all their branches. Many of these men, of my own knowledge, have never been within the walls of a medical college.

Now, with these existing facts, is there a man who would dare to say that such a person should have the right to go about imposing upon a community, pretending to be a healer of disease, or to allay human suffering, (often killing by neglect, if not by direct medication), simply because he is supposed to have a right to pursue such means of making money as he pleases? I say no, and every man, woman and child in Texas should join me in the dissention.

What is the remedy? We have had various bills formulated and presented for both our consideration and that of our legislators, but so far all have failed of their purpose. I do not wish to present a theory of my own, but desire to give a synopsis of the bill prepared by our new president of the State Association, Dr. Wilson, and recently unanimously adopted by that body at Houston. This bill seems long and complicated, but a careful study of it will bring out merit never yet presented in such a measure for Texas legislators' consideration. And as that meeting was a splendid representative body of the doctors of Texas, I shall lend it my earnest support.

This bill provides for a Medical Council of Texas, composed of the Attorney-General, State Health Officer, senior member of Board of Regents of the University, and the presidents of the three boards of examiners hereinafter provided for.

The duty of this council is to prepare questions and recommend applicants for examination, and finally review examinations and issue certificates.

It further provides for the Governor to appoint three boards of examiners, to be named the Texas State Board of Examiners, the Homeopathic Board of Examiners, and Eclectic Board of Examiners. These boards shall meet twice a year, examine such applicants as have been referred to them by the medical council, the questions to each board being the same except on therapeutics and practice.

There are many other provisions in the bill all tending to have a well qualified board and strict examinations, and providing for ample penalties for violation. The law will not affect those legally practicing medicine now.

I said in my beginning that the frequent discussions of this subject had been so thorough, and the conclusions recently reached so complete, that I could add but little to them, but that the most important work yet was before us, and which I now wish to present to you and trust at least to enlist every member of this Association in the work. How shall we secure such legislation as we need?

Every great evolution which has marked the different epochs in the history of ancient nations was caused only by long toil and earnest and continued effort on the part of those who desired such changes.

When this country freed itself from the oppression of Great Britain, it was through the interest and zeal of a few of our earlier settlers who banded themselves together to throw off the yokes of burden they had so long carried.

The effects of the great civil war are only the results of a sentiment springing from the minds of a few; and the words of Mrs. Harriet Beecher Stowe's Uncle Tom's Cabin set fire to that sentiment, which revolutionized this government and made a free State of a slave State.

No set of men, no party or association, will ever secure legislation simply by the asking; you must force your representatives to give consideration to your wants by creating a public demand for such legislation. Let us then as a united profession decide what we need and what we want, and then as a solid phalanx let us present our needs to the people whom we represent, and have them to demand the same of our legislators.

But you might say it is useless to endeavor to get political conventions to take any interest in our affairs. I first say that the needed medical legislation in this State is not alone our affair, but one which should concern every citizen in Texas, and it becomes our duty to impress this fact upon those with whom we associate.

Then how are we to begin? Let every doctor in Texas use his influence at home among his patrons, in his county conventions, and with his representative, to educate them to the importance of such a measure. The people do not read medical journals, nor attend medical associations, so it behoves us to change our tactics and place our wants before them.

You say you have no political influence? Well, probably you are right; but I say it does not require political influence in the sense that most people speak of political influence. Whom do you wish to influence? Your patrons and associates. Has the man who calls you into his home, where the little circle around his fireside constitutes his happiness, to administer to the physical needs of that child, no confidence in you? Will that man bring you into his home, and make you acquainted with the secrets of his family and family history, when he has no confidence in you? Will he call upon you, when threatened by contageous or epidemic disease, to protect his community, his family and himself, and still lack confidence in your ability and integrity? Will he allow you to usher in this world his beloved babe, care for its health and administer to it in its affliction, then finally, when you have done what you can and it becomes your duty to give the sad news that death approaches, and through the tender sympathies of your own heart attempt to console its parents in their hour of affliction, when he does not have uttermost confidence in your ability and honor?

Now you tell me that you are not able to go with this same man in your county convention, and there make known to him that your beloved profession stands in need of such laws as will include only those of known ability and integrity in its ranks; that such measures are not for your protection, but for the common protection of us all and for the good of the public health and welfare, and secure his support and influence? If so, he is indeed an ungrateful friend.

Now, gentlemen, I have not attempted to outline a law or frame a bill of my own; but after presenting the one recommended by our State Association, I have endeavored to urge upon you what I think is most needed to secure medical legislation in Texas. Let us each determine to do our duty and make some effort to secure for our profession that of which it has so long stood in need, and the lack of which has been the cause of us hearing such uncalled for and such unjust criticism of our profession, by our legal brothers, as well as by many of the laity. And if the profession of Texas will stop theorizing and writing bills to medical journals, and go to the people with their needs in such a manner as to prove our faithfulness to the cause, we will get what we have long been asking, and not until then.

